# Trial Forge Guidance 2: how to decide if a further Study Within A Trial (SWAT) is needed

**DOI:** 10.1186/s13063-019-3980-5

**Published:** 2020-01-07

**Authors:** Shaun Treweek, Simon Bevan, Peter Bower, Matthias Briel, Marion Campbell, Jacquie Christie, Clive Collett, Seonaidh Cotton, Declan Devane, Adel El Feky, Sandra Galvin, Heidi Gardner, Katie Gillies, Kerenza Hood, Jan Jansen, Roberta Littleford, Adwoa Parker, Craig Ramsay, Lynne Restrup, Frank Sullivan, David Torgerson, Liz Tremain, Erik von Elm, Matthew Westmore, Hywel Williams, Paula R. Williamson, Mike Clarke

**Affiliations:** 1 Health Services Research Unit, University of Aberdeen, Aberdeen, UK; 2 National Institute for Health Research Evaluation, Trials and Studies Coordinating Centre, Southampton, UK; 3 MRC North West Hub for Trials Methodology Research, Centre for Primary Care and Health Services Research, University of Manchester, Manchester, UK; 4 Department of Clinical Research, Basel Institute for Clinical Epidemiology and Biostatistics, University Hospital Basel and University of Basel, Basel, Switzerland; 5 GSK Medicines Research Centre, Stevenage, UK; 6 Health Research Authority, London, UK; 7 HRB-Trials Methodology Research Network, National University of Ireland Galway, Galway, Ireland; 8 Centre for Trials Research, College of Biomedical & Life Sciences, Cardiff University, Cardiff, UK; 9 Division of Acute Care Surgery, University of Alabama at Birmingham, Birmingham, USA; 10 University of Queensland Centre for Clinical Research, University of Queensland, Brisbane, Australia; 11 York Trials Unit, University of York, York, UK; 12 Public and patient representative, Aberdeen, UK; 13 School of Medicine, St Andrews University, St Andrews, UK; 14 Cochrane Switzerland, Institute of Social and Preventive Medicine (IUMSP), Lausanne University Hospital, Lausanne, Switzerland; 15 Centre of Evidence-Based Dermatology, Queen’s Medical Centre, Nottingham University Hospitals NHS Trust, Nottingham, UK; 16 MRC North West Hub for Trials Methodology Research, Department of Biostatistics University of Liverpool, Liverpool, UK; 17 Northern Ireland Methodology Hub, Queen’s University Belfast, Belfast, UK

## Abstract

The evidence base available to trialists to support trial process decisions—e.g. how best to recruit and retain participants, how to collect data or how to share the results with participants—is thin. One way to fill gaps in evidence is to run Studies Within A Trial, or SWATs. These are self-contained research studies embedded within a host trial that aim to evaluate or explore alternative ways of delivering or organising a particular trial process.

SWATs are increasingly being supported by funders and considered by trialists, especially in the UK and Ireland. At some point, increasing SWAT evidence will lead funders and trialists to ask: given the current body of evidence for a SWAT, do we need a further evaluation in another host trial? A framework for answering such a question is needed to avoid SWATs themselves contributing to research waste.

This paper presents criteria on when enough evidence is available for SWATs that use randomised allocation to compare different interventions.

## Introduction

The evidence available to inform many routine process decisions in randomised trials is thin or weak. This includes the evidence on how best to recruit participants [1], retain them [2], collect their data [3] or include them in decisions about the trial [4]. While evidence gaps in, say, the clinical management of diabetes might be expected to lead to a sustained and substantial research effort to fill them, similar effort has not materialised for trial methods research. Recruitment remains a major concern [5, 6] despite more than 25,000 new trials opening every year and needing to recruit participants [7]. Once recruited, there is also little evidence available to inform decisions about how to encourage trial participants to remain in the trial and, for example, to attend face-to-face measurement visits, which are a vital part of most trials [2]. Further, there is almost no evidence base to inform trial management decisions, including how to select sites, whether visiting them in person is worth it, or how to train staff [8].

The lack of trial process evidence contributes to research waste—for example, through poor recruitment, retention and data quality—and has been a feature of medical research for decades [9], with some suggesting that up to 85% of medical research spending is wasted [10]. However, much of the waste is avoidable [11] and research funders recognise the need to avoid it [12].

Trial Forge (http://www.trialforge.org) is an initiative that aims to improve the efficiency of trials, particularly by filling gaps in trial process evidence [13]. One way of improving the evidence base for trial process decisions is to do a Study Within A Trial (SWAT) [14], which is a ‘...self-contained research study that has been embedded within a host trial with the aim of evaluating or exploring alternative ways of delivering or organising a particular trial process’ [15]. For example, a SWAT could evaluate a new way of presenting information to potential participants as a way of improving trial retention, perhaps by being clearer about what taking part in the trial entails. Half of potential participants could be randomised to receive the new information while the other half receive the standard information. The effect of the new information on trial retention could be measured at the end of the trial or possibly part-way through if the trial has a long duration. Other interventions that could be evaluated in a SWAT include remote site training compared to face-to-face training, sending participants thank-you letters after attending trial visits and sending birthday cards to children in paediatric trials to improve retention. Any improvements that will arise from using an alternative approach for a particular process are likely to be modest but the combined effect of small improvements across many processes may well be substantial.

There is a growing repository of protocols for SWATs (http://bit.ly/20ZqazA) and Madurasinghe and colleagues have developed a reporting standard for recruitment SWATs, which are a priority for trial methodology research [16–18]. Moreover, major funders are taking the need for SWATs seriously as a vehicle for more efficient use of public resources. For example, the UK’s National Institute for Health Research Health Technology Assessment program (NIHR HTA) now highlights SWAT funding in all its trial funding calls and was the topic of a recent ‘HTA Director’s Message’ (https://www.youtube.com/watch?v=PoIE6xxK-pA). The Health Research Board Trial Methodology Research Network (HRB-TMRN) in Ireland also funds SWATs [19] and the Health Research Board encourages investigators to include a SWAT when applying for funding for both feasibility and definitive trial funding [20].

An important question to ask when thinking about undertaking SWATs is how to prioritise interventions for their first evaluation in a SWAT. A good example of a prioritisation process for unanswered questions for trial recruitment is the PRioRiTY project [18] (https://priorityresearch.ie). PRioRiTY 2 does the same for trial retention [21].

The scope of the work described here is what happens after the first evaluation. When evidence is available for an intervention or some aspect of the trial process, how should one decide if further evaluation is needed in another SWAT? Deciding whether a particular intervention needs further evaluation will always be a judgement. The objective of this Trial Forge guidance is to provide a framework for making this an informed judgement based on explicit criteria that most trialists and methodologists can agree with. We take a pragmatic stance about evidence generation: trial teams need enough evidence to know whether something is worth doing, no more and no less. The aim is to avoid wasting research effort evaluating interventions for which there is already good enough evidence for decision-making, allowing attention to re-focus on those interventions where important uncertainty still exists. This paper presents criteria for how to do this for SWATs that use randomised allocation to compare different interventions.

The guidance is written from the perspective of whether a single research team should do a further single evaluation of a SWAT in a single host trial as this is currently the most likely approach to doing a SWAT. Although we take a single SWAT perspective in this guidance, we expect it to apply equally well to SWATs done as part of a coordinated package of evaluations.

## Proposed criteria for making informed judgements about further SWAT evaluation

The main users of SWAT results will be members of trial teams. Funders of SWATs and trials are also likely to be interested. To make informed judgements, these users need to know what the accumulating evidence is for the effect of the SWAT on one or more relevant trial process outcomes (e.g. recruitment, retention), as well as the certainty for that evidence. They will want to know whether the evidence comes from evaluations done in contexts similar to their own. Finally, they will want to know how finely balanced the advantages and disadvantages of using the SWAT are, both for trial participants and the host trial.

Given the above, the five criteria we propose for deciding whether a further SWAT evaluation is needed are listed in Table 1. The aim of applying these criteria is to ensure that the need for a new evaluation is considered explicitly in light of what is already known about the intervention. Generally speaking, the more criteria that are met, the more likely we are to conclude that a new evaluation in a SWAT is appropriate. Conversely, if none of the criteria are met it is unlikely that a new evaluation would be appropriate.

**Table 1:** Should we do a further evaluation of the intervention in a SWAT?

The five proposed criteria for deciding whether the intervention needs another evaluation in a SWAT. The more criteria that are met, the more likely we are to conclude that further evaluation in a SWAT is appropriate.	
1. *GRADE*: the GRADE [22] certainty in the evidence for all key outcomes is lower than ‘high’.^a^	
2. *Cumulated evidence*: the cumulative meta-analysis shows that the effect estimate for each outcome essential to make an informed decision has not converged.^b,c^	
3. *Context*: the range of host trial contexts evaluated to date does not translate easily to the context of the proposed SWAT.^d^ For the proposed SWAT consider PICOT [23]:	
• P – is the population in the host trial so different from those already included that the current evidence does not provide sufficient certainty?	
• I – are the health interventions in the host trial so different from those already included that the current evidence does not provide sufficient certainty?	
• C – is the comparator in the host trial so different from those already included that the current evidence does not provide sufficient certainty?	
• O – is the SWAT outcome(s) so different to those used in the existing evaluations that that the current evidence does not provide sufficient certainty?	
• T – in the time since the existing evaluations were done, have regulatory, technological or societal changes made those evaluations less relevant?	
4. *Balance – participants*: the balance of benefit and disadvantage to participants in the host trial and/or the SWAT is not clear.^e^	
5. *Balance – host trial*: the balance of benefit and disadvantage to the new host trial is not clear.^f^	

Notes

^a^ A GRADE assessment of ‘high’ means that we are confident that the true effect lies close to the estimate of effect coming from the cumulative meta-analysis [24]. In Cochrane’s deliberations as to when to close a Cochrane Review (https://www.cochranelibrary.com/cdsr/doi/10.1002/14651858.ED000107/full), the collaboration chose not to require ‘high’ GRADE certainty in the evidence because it was felt that this may not always be achievable. Although we recognise the pragmatic nature of this, we recommend ‘high’ in our criteria because SWATs are usually simple studies for which it should be possible to generate high certainty evidence. We will, however, keep this criterion under review to consider whether it needs relaxing.

^b^ This is a judgement that depends on the behaviour of the effect estimates and on whether the confidence intervals include the threshold for an important benefit (or disadvantage). For example, if there is drift in the effect estimates of a meta-analyses but the confidence intervals around the estimates are consistently above what you think is an important benefit (or below a relevant disadvantage) then the cumulative meta-analysis can be judged to have converged despite movement in the effect estimates. For more on GRADE see http://www.gradeworkinggroup.org.

^c^ This is a judgement that depends on the behaviour of the effect estimates and on whether the confidence intervals include the threshold for an important benefit (or disadvantage). For example, if there is drift in the effect estimates of a meta-analyses but the confidence intervals around the estimates are consistently above what you think is an important benefit (or below a relevant disadvantage) then the cumulative meta-analysis can be judged to have converged despite movement in the effect estimates. For more on GRADE see http://www.gradeworkinggroup.org.

^d^ This is to provide reassurance about the applicability of the result to different types of trials. Care is needed to avoid a default position of insisting on an evaluation in every conceivable context. In other words, is there any reason to believe that the intervention would *not* work in your context given the contexts already studied? It is possible that evidence from SWATs will eventually splinter off to focus specifically on certain contexts but, for now, we suggest pooling evaluations of the same intervention because there are so few SWAT evaluations of any intervention and this pooling will provide a basic foundation on which to build.

^e^ Where there may be no conceivable benefit or disadvantage for participants, they should be considered as balanced.

^f^ A benefit might be that the host trial recruits faster, or its data quality is improved. Examples of disadvantages might be that there are added costs to the host trial, or that a new task is introduced into the workload of trial managers.

To illustrate the use of these criteria, we have applied them to examples from the Cochrane Review on strategies to improve trial recruitment [1] and the Cochrane Review on strategies to improve trial retention [2].

## Example 1: telephoning non-responders to trial invitations

### Background

Only two interventions in the 2018 version of the Cochrane Review for trial recruitment [1] have both high certainty for the evidence and a potential for widespread applicability. One of these is telephoning people who do not respond to postal invitations to take part in a trial, which is used in this example. (The other relates to optimising the patient information leaflet.) The Cochrane Review notes that the rating of high certainty is only for trials with low underlying recruitment of < 10% of eligible participants. If the evidence is to be applied to trials with higher underlying recruitment, the review authors suggested that the GRADE rating be reduced from high to moderate because of indirectness.

A trial team that includes people with lived experience of the illness or condition targeted is likely to consider information about the following essential when deciding whether a further evaluation of telephone reminders should form part of their recruitment strategy:

i. effect on recruitment
ii. cost
iii. participant irritation at receiving the telephone call

### Applying the five criteria

Table 2 summarises the results of the two telephone reminder trials and the overall estimate of effect.

**Table 2:** The cumulative effect estimates for the two telephone reminders compared to no reminder studies included in the updated Cochrane recruitment interventions review [1]

	Total number of participants	Intervention (n recruited/N invited)	Control (n recruited/N invited)	Baseline (control) recruitment rate	Effect estimate (95% CI)
**Nystuen, 2004** [25] (Telephoning people aged 16–66 years who had not responded to initial invitation by 2 weeks. Comparator was no call. Calls were made by research team. People were being recruited to a return to work trial for people on sick leave for > 7 weeks).	498	31/256	11/242	4.5%	8% (3%–12%)
**Wong, 2013** [26] (Telephoning people aged 50–70 years who had not responded to initial invitation by 4 weeks. Comparator was no call. Calls were made by research nurses. People were being recruited to a colorectal cancer screening trial).	952	59/480	35/472	7.4%	5% (1%–9%)
**Cumulative results** **(Nystuen + Wong)**	1450	90/736	46/714	6.0% (mean)	6% (3%–9%)

The GRADE rating of the certainty in the evidence is high

1. Both trials are scored as low risk of bias on the Cochrane Risk of bias tool

2. The results are consistent

3. The outcome was direct

4. The results are not imprecise; the confidence intervals are not too large and wholly on the side of benefit

5. There are too few trials for an assessment of publication bias and we have assumed that there is none

NOTE: the evidence for this intervention comes entirely from trials with low (< 10%) underlying recruitment. When applied to trials with higher recruitment we would downgrade the GRADE assessment because of Indirectness to moderate

Applying the criteria in Table 1:

1. *GRADE*. Data are available for recruitment only (two trials, n = 1450). The GRADE certainty in the evidence for the two trials in the review is high but is considered moderate for trials that do not have low (< 10%) underlying recruitment. *Criterion partially met* (the GRADE certainty in the evidence for all essential outcomes is lower than ‘high’).
2. *Cumulative evidence*. Data are available for recruitment only. There are only two trials and it seems too early to claim the cumulative meta-analysis has converged. *Criterion met* (the effect estimate for each essential outcome has not converged)*.*
3. *Context*. The PICOT for the available evidence is:
  P – One study was done in Norway in 2002–2003 and involved people aged 16–66 years who were sick-listed for > 7 weeks due to non-severe psychological problems or musculoskeletal pain. The second study was done in Canada in 2010 and involved people aged 50–70 years from family practice lists who were eligible for colorectal cancer screening.
  I – The host trial intervention in the Norwegian study was solution-focused sessions led by psychologists that were one-on-one or in groups and aimed to help people get back to work. The host trial interventions in the Canadian study were one of virtual colonoscopy, optical colonoscopy or faecal occult blood testing.
  C – The host trial comparator in the Norwegian study was usual care: written information from the social security office. The Canadian host trial was doing a head-to-head evaluation of three screening methods, so the three interventions mentioned above were also the comparators.
  O – Both studies measured recruitment to the host trial. Both host trials had low underlying recruitment.
  T – Mobile telephones have replaced home-based phones for many people and neither study explicitly includes mobile telephones.

Considering the above, leads to **Criterion partially met** (a new evaluation is likely to contain several elements in the PICOT that are importantly different to those in the two existing evaluations).

1. *Balance – participants.* There is little or no direct benefit to participants, although some may like being reminded about the trial. One potential disadvantage is that some participants may be irritated by the reminder call but what proportion would be irritated is unclear. *Criterion met* (the balance of benefit and disadvantage to participants in the new host trial and/or SWAT is not clear)
2. *Balance – host trial.* The benefit to the host trial is a small increase in recruitment if underlying recruitment is low but it is unclear what the benefit would be if underlying recruitment was higher. There is a potential disadvantage to the host trial of over-burdening trial staff with making the reminder telephone calls but the size of this disadvantage is unclear. *Criterion met* (the balance of benefit and disadvantage to those running the host trial is not clear)

Considering the responses across all five criteria leads us to conclude that further evaluation of telephone reminders is needed and especially where underlying recruitment is anticipated to be > 10%. The views of people with lived experience of the conditions targeted by host trials on receiving telephone reminder calls should be sought in future evaluations. More information on cost and the potential disadvantages for the host trial would also be welcome, as would evaluations that used mobile telephones.

**Fig. 1:**
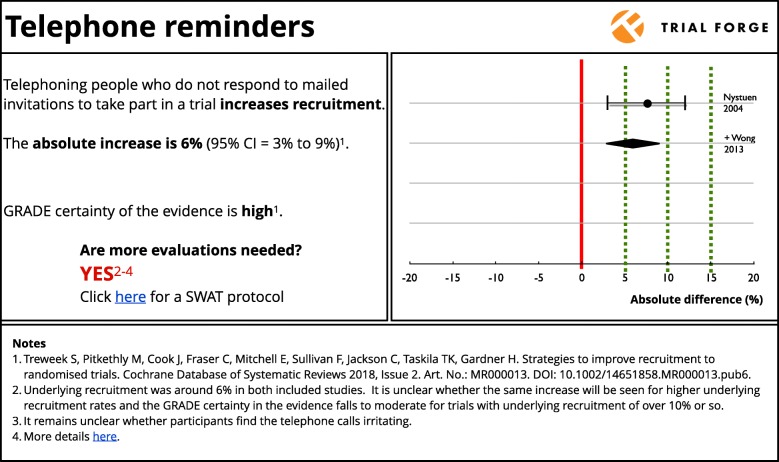
Summary of the cumulative evidence for the effect of telephone reminders on trial recruitment. The dotted lines represent decision thresholds of 0%, 5%, 10% and 15% that trialists can consider when deciding whether to use the intervention in their own trial

Figure 1 shows how the evidence with regard to telephone reminders for recruitment might be shown on the Trial Forge website. The cumulative meta-analysis in this summary shows four decision thresholds (absolute difference of 0%, 5%, 10% and 15%) that trialists can use when deciding whether they want to use the intervention in their own trial based on the current evidence. A trialist looking for a 10% or better increase in recruitment would probably decide that telephone reminders are not worth the effort, especially if underlying recruitment is not expected to be low. While a trialist expecting very low underlying recruitment might decide that any increase, even a small one, is worth having and plan their resource use accordingly. In both circumstances, the trialists would need to speculate on the balance of benefit to disadvantage.

## Example 2: monetary incentives to increase response rates to trial questionnaires

### Background

The 2013 Cochrane Review of interventions to improve trial retention [2] found that monetary incentives seem to improve response rates to trial questionnaires. A trial team that includes people with lived experience of the illness or condition targeted is likely to consider information about the following essential when deciding whether a further evaluation of financial incentives should form part of their retention strategy:

i. effect on questionnaire response rate (retention)
ii. cost
iii. participant irritation at receiving a small, unsolicited gift

### Applying the five criteria

Table 3 summarises the results of the three monetary incentives trials and the overall estimate of effect.

**Table 3:** The cumulative effect estimates for the three monetary incentives compared to no incentive studies included in the Cochrane retention interventions review [2]

	Total number of participants	Intervention (n recruited/N invited)	Control (n recruited/N invited)	Baseline (control) recruitment rate	Effect estimate (95% CI)
**Bauer, 2004** [27] (Sending $10 or $2 with invitations to return DNA sample (in mouthwash). Comparator was no money. People responding were a subgroup of a smoking cessation trial population).	300	77/200	34/100	34%	5% (−7% to 16%)
**Kenyon, 2005** [28] (Sending £5 voucher with invitations to return trial follow-up questionnaire. Comparator was no money. People responding were taking part in a trial to improve neonatal outcomes).	722	156/369	108/353	31%	12% (5%–19%)
**Gates, 2009** [29] (Sending £5 voucher with invitations to return trial follow-up questionnaire. Comparator was no money. People responding were taking part in a trial to improve neck injury outcomes).	2144	560/1070	493/1074	46%	6% (2%–11%)
**Cumulative results** **(Bauer + Kenyon + Gates)**	3166	793/1639	635/1527	37% (mean)	8% (4%–11%)

The GRADE rating of the certainty in the evidence is moderate

1. Only one of the three trials is scored as low risk of bias on the Cochrane Risk of bias tool; one was uncertain, the other high risk of bias. We considered this a serious limitation and downgraded 1 level

2. The results have some inconsistency in confidence intervals but not the direction of effect and on balance we decided not to downgrade

3. The outcome was direct

4. The results showed signs of imprecision but just for the smallest trial; the confidence intervals of the two larger trials are not too large and wholly on the side of benefit. We did not downgrade

5. There are too few trials for an assessment of publication bias and we have assumed that there is none

Applying the criteria in Table 1:

1. *GRADE*. Data are available for questionnaire response rates only (three trials, n = 3166). The overall GRADE certainty in the evidence is moderate. *Criterion met* (the GRADE certainty in the evidence for all essential outcomes is lower than ‘high’).
2. *Cumulative evidence*. Data are available for questionnaire response rates only. There are only three trials and it seems too early to claim that the cumulative meta-analysis has converged. *Criterion met* (the effect estimate for each essential outcome has not converged).
3. *Context*. The PICOT for the available evidence is:
  P – Two trials were done in the UK, one in 2002–2003 and the other in 2007–2008. The first involved women who had had a baby. The second UK study involved people aged > 18 years who attended emergency departments with a whiplash injury of < 6 six weeks’ duration. A third trial was done in the US in 2001 and involved smokers who wanted to stop.
  I – The host trial intervention in the 2002–2003 UK study was an antibiotic, while in the 2007–2008 UK study the host trial intervention was a book of advice about whiplash, with that advice being reinforced depending on the persistence of symptoms. The host trial intervention in the US study was a community-based program of public education, advice from healthcare providers, work-site initiatives and smoking cessation resources.
  C – The host trial comparator in the 2002/3 UK study was placebo and usual whiplash advice in the 2007/8 UK study. The host trial comparator in the 2001 study was no community-based smoking cessation program.
  O – All studies measured retention to the host trial. All three host trials had underlying retention < 50%.
  T – The most recent of these studies was done in 2007–2008 so inflation and other societal changes may affect the attractiveness of the amounts paid.

Considering the above, leads to **Criterion partially met** (a new evaluation is likely to contain several elements in the PICOT that are importantly different to those in the three existing evaluations).

1. *Balance – participants.* There is modest financial benefit to participants who receive the incentive. The potential disadvantage of a participant feeling coerced to provide questionnaire data seems low given the size of financial incentive being offered in these trials (US$10 or less) although whether these small amounts are perceived as insulting or irritating is unclear. *Criterion partially met* (the balance of benefit and disadvantage to participants in the new host trial and/or SWAT is not clear).
2. *Balance – host trial.* The benefit to the host trial is a modest increase in response rates. The potential disadvantage to the host trial of the costs of providing the incentives is quantifiable. Workload may be increased (e.g. someone has to manage vouchers or other incentives) but this is unlikely to be much larger than the work needed anyway to send out questionnaires. *Criterion not met* (the balance of benefit and disadvantage to those running the host trial is clear and can be estimated for each trial depending on the size of the incentive).

Considering the responses across all five criteria leads us to conclude that further evaluation of financial incentives is needed with priority given to evaluation in trials expected to have underlying retention > 50%. The views of people with lived experience of the conditions targeted by host trials on receiving small, unsolicited payments should be sought in future evaluations. Future randomised evaluations should ensure that they are assessed as at low risk of bias on the Cochrane Risk of Bias tool [30] to move the GRADE assessment from moderate to high.

**Fig. 2:**
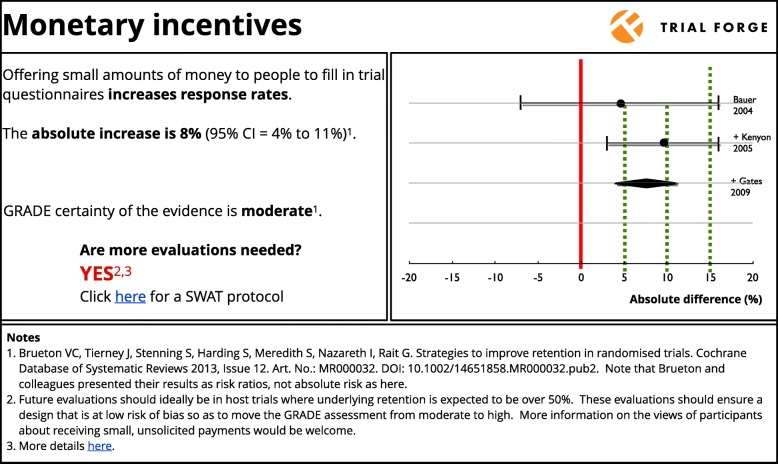
Summary of the cumulative evidence for the effect of monetary incentives on trial retention. The dotted lines represent decision thresholds of 0%, 5%, 10% and 15% that trialists can consider when deciding whether to use the intervention in their own trial

Figure 2 shows how Trial Forge might summarise the evidence with regard to monetary incentives for retention.

## Discussion

Trial Forge is an initiative to strengthen the evidence base for trial process decision-making, as one step towards improving the effectiveness and efficiency of those processes. SWATs are an important way of contributing to that evidence base. However, in order to minimise research waste arising from the SWATs themselves, their designers need to be confident that enough evidence is not already available from evaluations of a given intervention to support good, evidence-informed decisions.

The five criteria shown in Table 1 provide a basis to determine whether this is the case. Although this approach requires judgement, it provides a transparent mechanism for deciding whether the GRADE assessment of the certainty of the evidence, cumulative meta-analysis, host trial contexts and balance of benefit and disadvantage suggest that there is merit in evaluating the intervention in more SWATs, or whether there is already enough information to support evidence-informed decision-making about the relevant trial process. It also provides a way to frame and track discussion between researchers on particular SWATs—recognising that there will be disagreements but providing clarity about these disagreements and subsequent decision-making. Moreover, using this approach will help to identify and prioritise SWATs where there is existing but insufficient evidence and the type of host trials that should be targeted to build the evidence base. The criteria can also be used with decision thresholds (e.g. benefits of 5%, 10%, 15% or more) to help people decide whether they want to use the intervention based on the existing evidence even if more evaluations are needed.

We will pilot this technique and the five criteria for those SWATs promoted through Trial Forge, making clear statements for these evaluations akin to those given above for the two examples. We expect that the technique will be refined and improved over time but, for now, the approach provides a starting foundation. Some areas that need work are mentioned below as limitations. The criteria might also be linked to the SWAT repository (http://bit.ly/20ZqazA), to improve the accessibility of SWAT results and ongoing SWAT evaluations. Showing that the criteria support a further evaluation of an intervention in a SWAT is also likely to be helpful to those deciding about applications for funding of new SWAT evaluations by providing reassurance about the need for the work and its contribution to the body of evidence.

There are some limitations. The sparseness of the trial process evidence base means that it is currently unlikely that applying the five criteria to any body of evidence will lead to a decision not to start another evaluation. We did want to include an example that would have shown the criteria concluding that more evaluations were unnecessary but the current paucity of research into trial processes means that we could not find one. In addition, the criteria have been developed by a group of SWAT enthusiasts who are based mainly in the UK or Ireland. Others may prefer different criteria and we hope that this paper will stimulate discussion and lead to refinements as these and other criteria are applied. Another limitation is the potential for publication bias. Anecdotally, we know that some SWATs are done but not published, which means our evidence summaries and judgements could suffer from publication bias. As others have noted [31], it is extremely difficult to be certain that publication bias is absent but by including GRADE, our criteria do include an explicit consideration of the potential for publication bias. Applying our criteria systematically across many SWAT interventions will also need resources. Finding these might be a challenge but our hope is that by demonstrating the value of the criteria in reducing research waste by highlighting when further evaluations of a SWAT are (or are not) needed will make it easier to secure resources in the future.

The most troubling limitation is likely to relate to the third criterion and the issue of context, which is no less thorny in SWATs than it is in the host trials in which they sit. We suggest a PICOT framework to consider contextual factors and there may be a need for additional factors to be considered. For example, our criteria do not explicitly dwell on the behavioural theory or mechanism of action behind a SWAT intervention and whether these theories and mechanisms still apply outside the context in which the intervention was developed. Our criteria may need to change, especially as bodies of SWAT evidence get larger. We welcome suggestions for the key variables needed by trial teams and others to make judgements about context, which can then be considered for inclusion in the *Context* criterion.

Finally, in a spirt of pragmatism about evidence generation, we recognise that less than perfect might be good enough and certainly better than no evidence at all. This may mean that the most efficient way of approaching the limited time and money available for evidence generation about trial processes may be to focus on whether something clears a threshold that makes it worth doing, rather than having a precise estimate of its effect. There would be little to gain from pursuing perfection if it will not change decisions. If we want to avoid wasting resources and participant goodwill, we need to think carefully about when enough is enough.

## Data Availability

Not applicable.

## Abbreviations

ELICIT: EvaLuation of Interventions for informed Consent for randomIsed controlled Trials
GRADE: Grading of Recommendations, Assessment, Development and Evaluations
HRB: Health Research Board
HTA: Health Technology Assessment
NIHR: National Institute for Health Research
PICOT: Population–Intervention–Comparator–Outcome–Time
PRioRiTy: Prioritising Recruitment in Randomised Trials
START: Systematic Techniques for Assisting Recruitment to Trials
SWAT: Study Within A Trial
TMRN: Trial Methodology Research Network
